# A Rhamnogalacturonan Acetylesterase Effector FsRGAE1 Enhances the Virulence of *Fusarium sacchari* by Localizing to the Nucleus and Suppressing Plant Immunity

**DOI:** 10.3390/jof12090638

**Published:** 2026-08-26

**Authors:** Huifang Li, Shuai Xu, Ying Chen, Han Zhang, Ye Tang, Yuetian Li, Shenghua Xiao, Qin Hu

**Affiliations:** College of Agriculture, Guangxi Sugarcane Bio-Breeding Laboratory, State Key Laboratory of Conservation and Utilization of Subtropical Agro-Bioresources, Guangxi Key Laboratory of Sugarcane Biology, Guangxi University, Nanning 530004, China; lihuifang_666@163.com (H.L.);

**Keywords:** *Fusarium sacchari*, Pokkah Boeng disease, effector, rhamnogalacturonan acetylesterase, immunity suppression

## Abstract

*Fusarium sacchari* is one of the major pathogenic fungi that cause sugarcane Pokkah Boeng disease (PBD). Effectors play pivotal roles in *F*. *sacchari*–sugarcane interaction; thus, characterizing these effectors is essential for elucidating the molecular mechanisms underlying *F. sacchari* pathogenicity and for developing effective strategies to control PBD. However, only a limited number of effectors have been functionally validated to date. Here, we report FsRGAE1, a candidate effector protein from *F. sacchari* predicted to encode a rhamnogalacturonan acetylesterase (RGAE). FsRGAE1 exhibits high expression during the early stages of infection and maintains relatively elevated expression levels throughout the *F. sacchari*–sugarcane interaction. Targeted deletion of the FsRGAE1 gene in *F. sacchari* had no discernible impact on mycelial growth, conidiation, or carbon-source utilization, yet it significantly attenuated fungal virulence. FsRGAE1 possesses both a signal peptide conferring secretory capacity and a transit peptide enabling its translocation into the host cytoplasm and nucleus. Using the *Agrobacterium tumefaciens*-mediated transient expression system in *Nicotiana benthamiana*, FsRGAE1 was confirmed to suppress cell death induced by Bcl-2-associated X protein (BAX), as well as ROS accumulation and callose deposition, and its nuclear localization is indispensable for this immunosuppressive activity. Collectively, these findings indicate that FsRGAE1 promotes *F*. *sacchari* virulence by suppressing host immune responses in a nuclear localization-dependent manner, providing new insights into effector-mediated *F*. *sacchari* pathogenesis and potential target for resistance breeding in sugarcane.

## 1. Introduction

Sugarcane is China’s most important sugar crop, contributing over 80% of national sugar production. As a strategic agricultural resource critical to national sugar security, ensuring a stable and reliable sugar supply constitutes a key governmental priority [1,2]. Sugarcane cultivation is highly concentrated in southern China, with Guangxi Zhuang Autonomous Region serving as the country’s principal production base. Pokkah Boeng disease (PBD) has been reported in virtually all countries where sugarcane is commercially cultivated and ranks among the most destructive fungal diseases affecting sugarcane yield. The predominant causal agent in China is the *Fusarium* fungal complex, including *Fusarium fujikuroi*, *F. sacchari*, *F. oxysporum*, *F. verticillioides*, *F. proliferatum*, and *F. subglutinans*, and *F*. *sacchari* [3]. This pathogen is a member of the genus *Fusarium* and infects sugarcane by impairing leaf photosynthesis and apical shoot growth. In the early stages of infection, the yellowing or chlorosis of young leaves is observed, with some leaves exhibiting red specks. As the symptoms develop, the leaves begin to crumple and twist. In the most severe cases, the top of the plant rots, which is termed top rot or stalk rot, and even complete plant death has been observed, causing substantial yield losses [3].

Over the past two decades, research into plant immunity has advanced substantially [4]. The foundational conceptual framework of pattern-triggered immunity (PTI) and effector-triggered immunity (ETI) has elucidated key mechanisms by which plants recognize pathogen-associated molecular signatures and mount effective defense responses. To successfully establish infection, pathogens must either evade host immune surveillance or actively suppress these defense pathways. Subsequently, pathogens may adapt through effector mutation, diversification, functional redundancy, or even loss of effectors to circumvent or suppress ETI. These dynamic interactions among PTI, ETS, and ETI collectively constitute the widely referenced “zig-zag” model of plant–pathogen co-evolution [5]. Crucially, the evolutionary core of this interaction centers on an ongoing molecular arms race between plant R genes and pathogen effectors. Effectors thus serve as pivotal virulence determinants for pathogens, and a comprehensive understanding of their structure, function, and mode of action is indispensable for deciphering the molecular basis of pathogen–host interactions.

At present, genomics and transcriptome sequencing are widely used to identify candidate effector genes in host–fungus interactions. For example, in the wheat leaf rust pathogen *Puccinia triticina*, long-read sequencing (LRS) with the FALCON/FALCON-Unzip (v4.1.0) pipeline generated the first LRS-based de novo genome assembly. Comparative genomic analysis across six isolates with divergent virulence profiles identified 38, 31, and 37 candidate avirulence genes for *AvrLr26*, *AvrLr2a*, and *AvrLr3ka*, respectively [6]. In the soil-borne pathogen *Verticillium dahliae*, nanopore sequencing produced a high-quality genome assembly and annotation, predicting 854 secreted proteins and 128 effectors [7]. In *Phytophthora ramorum*, PacBio LRS of the aggressive isolate CDFA1418886 yielded a high-quality 60.5-Mb assembly (ND886). This revealed 21 and 25 CRN effectors and 393 and 394 RXLR effectors in haplotypes A and B, respectively, including 24 and 25 newly predicted RXLR effectors. These findings provide novel insights into effector heterozygosity and the genomic architecture of *P. ramorum* [8].

In *Fusarium sacchari*, one of the causative agents of Pokkah Boeng disease in sugarcane, 316 candidate secreted effector proteins (CSEPs) were predicted from the complete genome sequence, and a foundational framework for investigating the roles of effector molecules in *F*. *sacchari*–sugarcane interactions has been established [9]. Compared with effector proteins from well-studied model pathogens such as *Magnaporthe oryzae*, *Phytophthora sojae*, and *Sclerotinia sclerotiorum,* whose identification and functional characterization have been extensively investigated, those from *F. sacchari* remain largely uncharacterized. Functional annotation and mechanistic insights into their interactions with the sugarcane host are severely limited, with only a few effectors, such as FsMEP1, FsEG1 and FsP4H1, experimentally validated to date [10,11,12]. This substantial knowledge gap critically constrains our understanding of the molecular basis of *F. sacchari*–sugarcane pathogenesis and impedes the development of effective strategies for managing PBD.

In this study, we identified FsRGAE1, a candidate effector in *F. sacchari* predicted to be a rhamnogalacturonan acetylesterase (RGAE), and characterized its role in *F. sacchari*–sugarcane interaction. We discovered that FsRGAE1 has secretory activity and can be localized to the *Nicotiana benthamiana*’s nucleus to inhibit the immunity responses, and the knocking out of FsRGAE1 significantly reduces the pathogenicity of *F. sacchari* on sugarcane. Our findings provide novel insights into *F. sacchari* pathogenesis and highlight potential targets for disease-management strategies.

## 2. Materials and Methods

### 2.1. Plant Materials, Fungal Strains, and Growth Conditions

This study employed the PBD-susceptible modern hybrid sugarcane variety “Zhongzhe No. 1” (ZZ1). Healthy, semi-mature sugarcane stems were surface-sterilized and planted in a substrate composed of humus soil and vermiculite (2:1, *v*/*v*), and grown in a greenhouse on the campus of Guangxi University at 30 °C and 70% relative humidity under a 14 h light/10 h dark photoperiod; *Fusarium sacchari* inoculation experiments were conducted on the leaves of sugarcane seedlings at the five-leaf stage. *Nicotiana benthamiana* plants used in this study were provided by our laboratory and cultivated in an artificial climate chamber maintained at 28 °C and 60% relative humidity under a 14 h light/10 h dark photoperiod.

The *Fusarium sacchari* wild-type strain CNO-1 (GenBank: JALNLS000000000.1) was cultured at 28 °C on potato dextrose agar (PDA) medium at 28 °C in a chamber. *Escherichia coli* DH5α was cultured at 37 °C. *Agrobacterium tumefaciens* strains GV3101 and AGL1 were employed for *Agrobacterium*-mediated transient expression in *N. benthamiana* and fungal transformation, respectively. *F. sacchari* knockout mutants were maintained on PDA supplemented with 50 μg/mL hygromycin, whereas complementary mutants were grown on PDA containing 50 μg/mL geneticin. The *Botrytis cinerea* strain B05.10 was cultured on PDA at 25 °C in a chamber for disease resistance assays. The yeast strain YTK12 used to validate the secretion function of signal peptides was stored in our laboratory and grown on YPDA medium at 30 °C.

### 2.2. Identification and Bioinformatics Analysis of FsRGAE1

The predicted coding sequence of *FsRGAE1* was identified according to our previous work, and cloned from the cDNA of *F. sacchari*. Conserved protein domains were identified using InterPro (https://www.ebi.ac.uk/interpro/search/sequence/; accessed on 8 October 2024). Signal peptides (SPs) were predicted with SignalP-6.0 (https://services.healthtech.dtu.dk/services/SignalP-6.0/; accessed on 8 October 2024). Phylogenetic analysis of FsRGAE1 alongside other RGAE homologs was conducted using the neighbor-joining method implemented in MEGA12. Multiple-sequence alignments were performed using DNAMAN software 9.0. Homology-based protein structure modeling was carried out using AlphaFold3 (https://alphafoldserver.com/; accessed on 8 October 2024) with default parameters, employing AaRGAE (UniProt ID: Q00017) as the structural template [13]. All primers used in this study are listed in Table S1.

### 2.3. RNA Extraction and RT-Quantitative PCR Analysis Inoculate

Total RNA was extracted from sugarcane leaves inoculated with *F. sacchari* strain CNO-1 at 0 (corresponding to CNO-1 mycelia prior to inoculation), 12, 24, 48, 72, 96, 120, and 144 h post-inoculation, using the Eastep Super Total RNA Extraction Kit (Promega, Shanghai, China). First-strand cDNA synthesis and RT-qPCR analysis were performed as previously described [11]. The reference genes *Fvactin*, *ScGADPH*, and *NbEF1α* were used as internal controls for *F. sacchari*, sugarcane, and *N. benthamiana*, respectively [10]. Relative gene expression levels were calculated using the 2^−ΔΔCt^ method [14]. Mean values and standard deviations were derived from at least three independent biological replications. Primers used in this study are listed in Table S1.

### 2.4. Yeast Signal Sequence Trap Assays

The yeast signal sequence trap system was employed to validate the putative function of the FsRGAE1 signal peptide as previously described [15]. The coding sequence encoding the predicted FsRGAE1 signal peptide was cloned and fused in-frame to the *EcoR*I and *Xho*I linearized pSUC2 vector, which carries a truncated invertase (SUC2) gene lacking its native signal peptide, generating SPFsRGAE1-pSUC2. The recombinant construct *SPFsRGAE1-pSUC2,* along with control constructs (negative controls, empty pSUC2 and SPMg87-pSUC2; positive control, *SPAvr1b-pSUC2*), was introduced into the yeast strain YTK12. All transformed yeast strains were individually cultured on YPDA medium, CMD-W (tryptophan-deficient) medium and YPRAA medium, with raffinose serving as the sole carbon source. Secretion of invertase was assessed by monitoring the growth of the yeast strains on the YPRAA medium. Invertase activity was further quantified by monitoring the reduction of 2,3,5-triphenyltetrazolium chloride (TTC) to insoluble red formazan as previously described [16].

### 2.5. Subcellular Localization Assay

The coding sequences of *FsRGAE1* and *∆SPFsRGAE1* (lacking signaling peptide regions) were amplified and individually inserted into the *BamH*I and *Kpn*I linearized PBI121-EGFP and pCambia1300-NES-GFP vectors, respectively, to generate C-terminal fused GFP constructs (*FsRGAE1-GFP* and *∆SPFsRGAE1-GFP*), and N-terminal fused NES (nuclear export signal) and C-terminal fused GFP constructs (*NES-FsRGAE1-GFP* and *NES-∆SPFsRGAE1-GFP*). All constructs were introduced into *Agrobacterium tumefaciens* strain GV3101 for transient expression in *N. benthamiana*. *A. tumefaciens* suspensions (OD_600_ = 0.8) were infiltrated into the abaxial surface of leaves from 4- to 5-week-old *N. benthamiana* plants. Epidermal cells were imaged 60 h post-infiltration using an Olympus FV3000 confocal laser scanning microscope (Olympus Corporation, Tokyo, Japan). Prior to imaging, plasmolysis was induced by incubating leaf samples in 0.8 M mannitol solution for 15 min [17]. The plasma membrane marker 35S::AtPLC2-RFP was co-expressed as a reference [18].

### 2.6. Generation of F. sacchari Knockout and Complementation Mutants

The *FsRGAE1* gene knockout (*ΔFsRGAE1*) and complementation (*ComΔFsRGAE1*) mutants were constructed using the *Agrobacterium*-mediated homologous recombination knockout and complementation strategy as previously described [11]. The positive transformants were screened on the PDA medium with 50 μg/mL hygromycin B or 50 μg/mL geneticin for gene deletion and complementation, respectively, and subsequently verified through PCR using the appropriate primer pairs. Primers used in this study are listed in Supplementary Table S1.

To investigate the role of FsRGAE1 in mycelial growth and morphology of *F. sacchari*, spore suspensions (1 × 10^5^ spores/mL) of the wild-type strain CNO-1, the knockout mutant *ΔFsRGAE1*, and the complemented strain *ComΔFsRGAE1* were individually inoculated at the center of plates containing media supplemented with various carbon sources. Colony morphology and radial growth rates were evaluated on days 3, 6, and 9.

### 2.7. Pathogenicity Tests

Pathogenicity assays of the *FsRGAE1* knockout and complemented mutants were conducted as previously described [12]. Wild-type CNO-1, *ΔFsRGAE1*, and *ComΔFsRGAE1* strains were pre-cultured on PDA medium for seven days. Then, 5 mm mycelial colonized agar plugs were inoculated onto the leaves of ZZ1 six-leaf-stage seedlings. The inoculated leaves were placed in plastic containers lined with moist filter paper and cultured for 7 days at 28 °C under a photoperiod of 16 h light/8 h dark. For each experiment, at least 9 leaves were inoculated, and a minimum of three biological replicates were performed. Seven days post-inoculation, necrotic lesion areas on the inoculated leaves were measured. The lesion tissue (samples taken from the leaf 2.5 cm away from the initial inoculation site) was excised, surface-disinfected, and transferred to PDA medium to assess fungal recovery. For each experiment, at least 3 lesion leaves were excised, and a minimum of three biological replicates were performed. The fungal biomass of *F. sacchari* in sugarcane cells was quantified using DNA-based quantitative PCR, as previously described [11]. For each inoculation treatment, six independent DNA samples were analyzed, and the entire experiment was conducted with three biological replicates. Primers used in this study are listed in Table S1.

### 2.8. Agrobacterium-Mediated Transient Expression in N. benthamiana

To investigate the role of FsRGAE1 in modulating host immune responses, the full-length coding sequences of *FsRGAE1*, *∆SP-FsRGAE1*, GFP, and BAX (BCL-2-associated X protein) were amplified and individually cloned into the pGR107 vector with a 3× HA tag at C-terminal, using the CloneExpress Ultra One Step Cloning Kit (Vazyme, Nanjing, China). All resulting constructs were introduced into *A. tumefaciens* GV3101 and transiently expressed in 4-week-old *N. benthamiana* leaves as previously described [19]. GFP and BAX served as negative and positive controls, respectively. Cell-death symptoms in *N. benthamiana* leaves typically appeared 5 to 7 days post-inoculation.

### 2.9. Botrytis cinerea Inoculation Assay

*Botrytis cinerea* inoculation assay on *N. benthamiana* leaves was conducted as previously described [11]. Briefly, the leaves transiently expressing the target proteins were collected at 24 h after agroinfiltration, and their petioles were wrapped with sterile-water-moistened absorbent cotton. *B. cinereal* strain B05.10 was pre-cultured on PDA medium at 25 °C for 7 days. A 5 mm diameter mycelial plug was excised from the edge of a mature colony and placed directly onto the agroinfiltrated leaf area. Inoculated leaves were placed in containers lined with moist filter paper and incubated at 25 °C under a 16 h light/8 h dark photoperiod. Lesion development was monitored daily, and lesion areas were measured and photographed over a period of 3 to 5 days.

### 2.10. Determination of ROS Burst and Callose

ROS and callose accumulation in *N. benthamiana* leaves were visualized using 3,3′-diaminobenzidine (DAB) staining and aniline blue staining, respectively, as previously described [20]. H_2_O_2_ measurements were carried out according to the manufacturer’s instructions using a micro hydrogen peroxide detection kit (Solarbio, Beijing, China). Callose deposition was calculated using the software ImageJ v1.52.

### 2.11. Statistical Analysis

Data were analyzed using Excel. Statistical analysis was performed using Student’s *t*-test (*, *p* < 0.05; **, *p* < 0.01). Error bars represent standard deviation (SD) of at least three biological replicates (the exact number of biological replicates is indicated in the corresponding figure legends).

### 2.12. Accession Numbers

Sequence data reported in this article are available in the Sol Genomics Network database (https://solgenomics.net/organism/Nicotiana_benthamiana/genome/; accessed on 13 February 2025) under the following accession numbers: *NbPR1a*, Niben101Scf04053g02006.1; *NbPR2*, Niben101Scf01934g02004.1; *NbHSR203J*, Niben101Scf05283g00016.1; and *NbLOX*, Niben101Scf01434g03006.1.

## 3. Results

### 3.1. Identification and Structural Analysis of FsRGAE1

**Figure 1:**
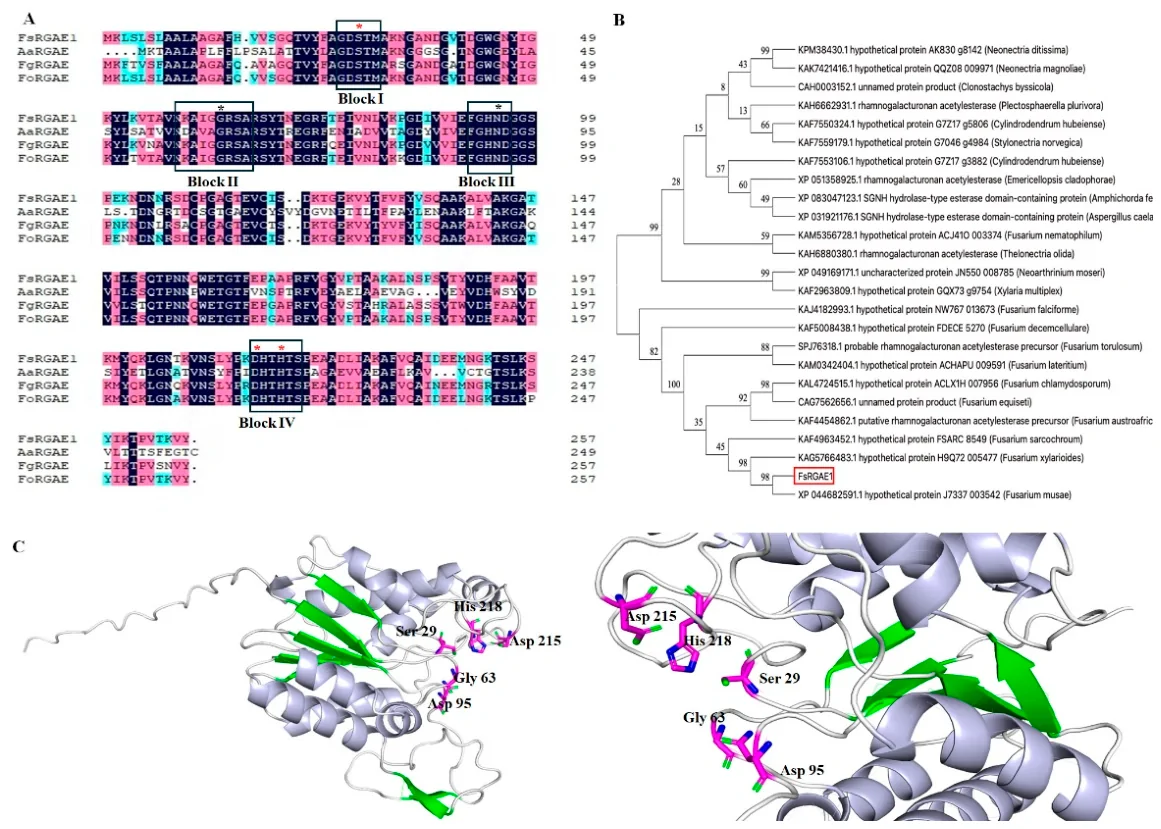
Identification and structural analysis of FsRGAE1. (**A**) Multiple-sequence alignment of FsRGAE1 and RGAE proteins of *Alternaria alternata* (AaRGAE), *Fusarium graminearum* (FgRGAE), and *Fusarium oxysporum* (FoRGAE). Four conserved sequence blocks (I, II, III and IV) and the characteristic residues contained in each conserved block are indicated in black boxes and stars, respectively. The constituent residues of the catalytic triad are indicated by red stars. (**B**) Phylogenetic analysis of FsRGAE1 protein and its orthologous proteins from other phytopathogenic fungi using MEGA12.0 with the neighbor-joining method with 1000 bootstrap replicates. The red box indicates the FsRGAE1 protein. (**C**) AlphaFold3-based structural prediction of FsRGAE1 reveals an α/β/α mezzanine fold and identifies the catalytic pocket residues within the SGHN motif.

Based on prior identification of candidate effector proteins from *F. sacchari*, a gene encoding a CE12 family rhamnogalacturonan acetylesterase (RGAE) was cloned from the cDNA of *F. sacchari* and designated *FsRGAE1* (Figure S1). The coding sequence of FsRGAE1 comprises 774 nucleotides, encoding a 257-amino-acid protein that features an N-terminal signal peptide (residues 1–20) and a predicted SGNH hydrolase catalytic domain. This domain is defined by four highly conserved sequence motifs (Blocks I–IV), with the GDS(L) motif located in Block I. Catalytic activity is strictly dependent on the invariant presence of four key residues, including Ser (S) in Block I, Gly (G) in Block II, Asn (N) in Block III, and His (H) in Block IV, forming the eponymous “SGNH” motif (Figure 1A). To investigate the evolutionary relationship between FsRGAE1 and other fungal RGAE proteins, a phylogenetic tree was constructed using the neighbor-joining method. The analysis revealed that RGAE homologs are widely distributed among phytopathogenic fungi, and FsRGAE1 shares its closest orthologous relationship with the RGAE protein from *Fusarium musae* (Figure 1B). The overall three-dimensional structure of FsRGAE1, predicted by AlphaFold3, adopts the canonical SGNH hydrolase α/β/α fold and retains the conserved catalytic residues characteristic of the SGNH family (Figure 1C).

### 3.2. FsRGAE1 Is Expressed During the Early Stage of Infection

**Figure 2:**
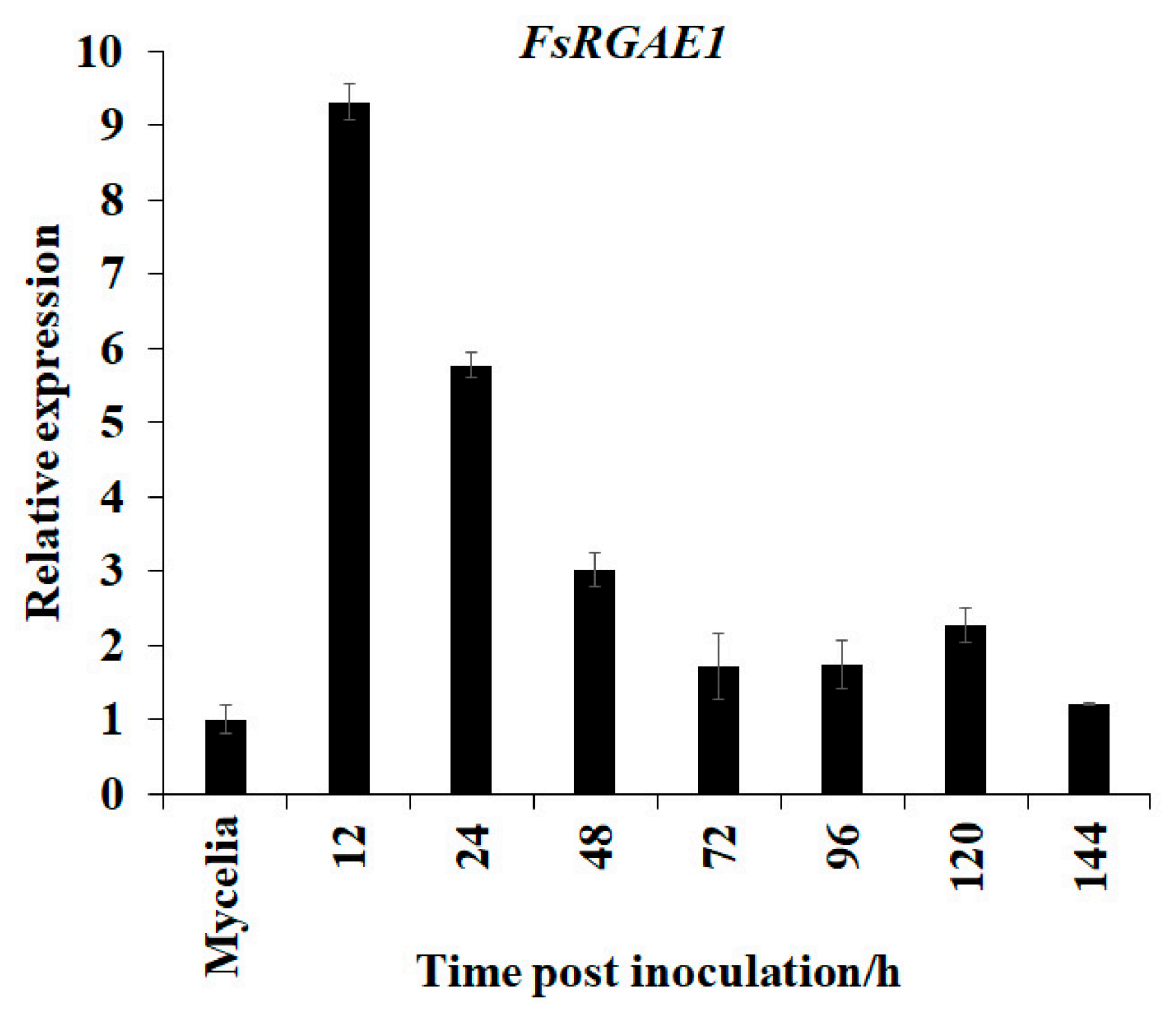
*FsRGAE*1 is expressed during the early stage of infection. Expression levels of FsRGAE1 in the leaves of sugarcane cultivar ZZ1 inoculated with *F. sacchari* strain CNO-1 at different stages of infection. Total RNA was extracted from mycelia or infected sugarcane leaves at 12, 24, 48, 72, 96, 120 and 144 h post inoculation. Values are the means ± SD; *n* = 3.

To elucidate the potential functional role of FsRGAE1 during *F. sacchari* infection of sugarcane, we examined its expression profile in sugarcane leaves at multiple time points post-inoculation using quantitative real-time PCR (qRT-PCR). The results revealed that *FsRGAE1* transcript levels were significantly upregulated following inoculation, peaking at 12 h post-inoculation (hpi), and subsequently declined gradually throughout later stages of infection, yet remained consistently higher than those observed in *F. sacchari* mycelia (Figure 2). These findings suggest that *FsRGAE1* is a canonical early-responsive gene and likely plays a potential role during the initial phase of pathogen infection.

### 3.3. The Signal Peptide of FsRGAE1 Possesses Secretory Function

**Figure 3:**
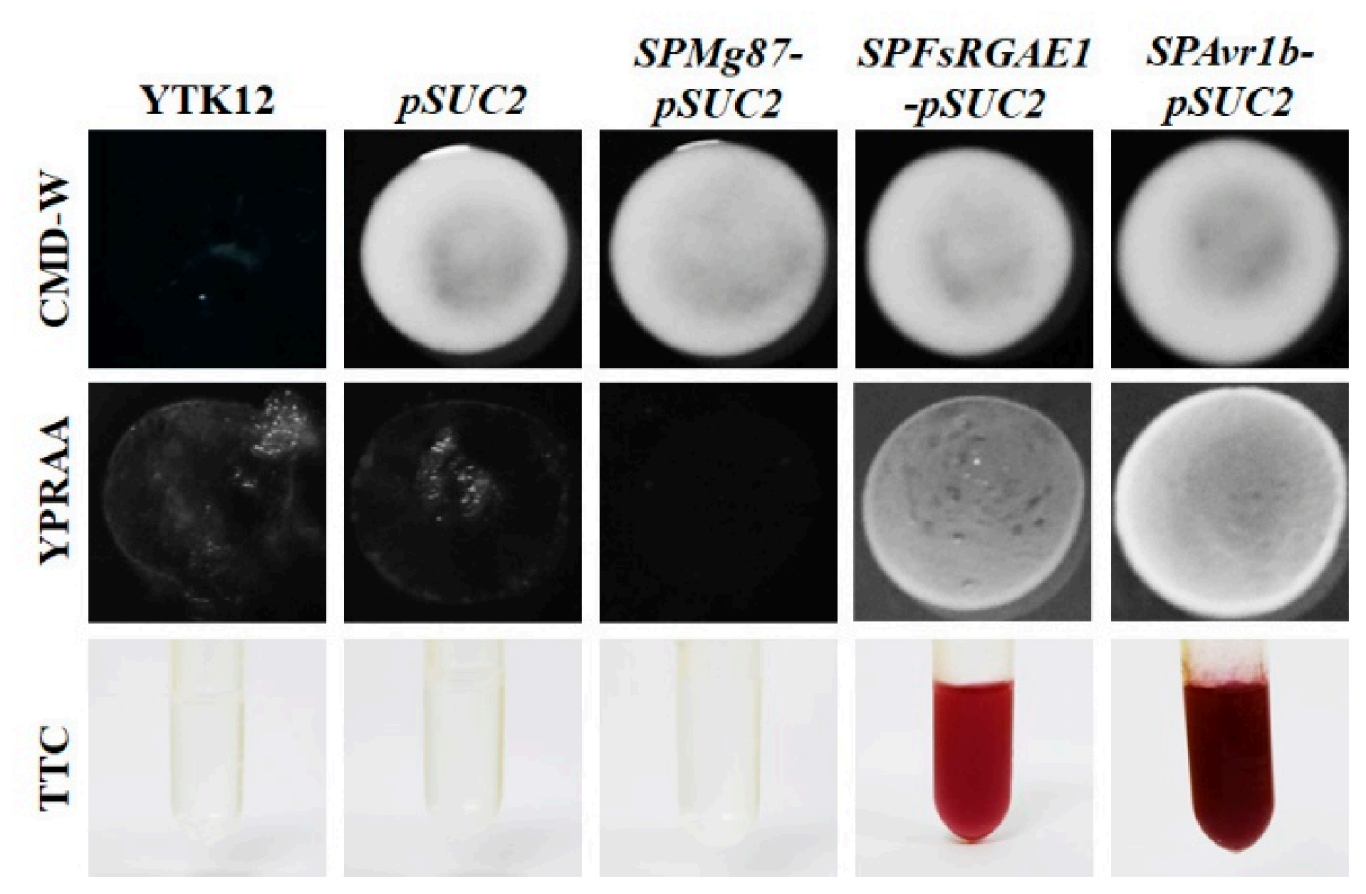
Secretion functional validation of the predicted signal peptide of FsRGAE1. The yeast strain YTK12 expressing FsRGAE1-pSUC2 grew on both CMD-W and YPRAA plates and produced a red colorimetric reaction. The yeast strain YTK12 expressing SPAvr1b-pSUC2 served as the positive control, whereas the yeast strains YTK12 expressing SPMg87-pSUC2 or the empty vector pSUC2 served as negative controls.

To verify the secretory function of the FsRGAE1 signal peptide, we performed a Yeast Signal Peptide Screen Trap (YSST) assay coupled with 2,3,5-triphenyltetrazolium chloride (TTC)-based catalytic detection. *SPMg87-pSUC2* (25 bp in the N-terminal of *Magnaporthe oryzae* protein Mg87) was used as a negative control, while *SPAvr1b-pSUC2* (a secreted signal peptide of Avr1b from *Phytophthora sojae*) was used as a positive control. As shown in Figure 3, all YTK12 strains carrying pSUC2 or pSUC2-recombinant plastids grew robustly on the sucrose-containing CMD-W medium, confirming successful plasmid transformation into the yeast host. Subsequent screening was conducted on YPRAA medium (raffinose-containing), where growth was observed exclusively for YTK12 strains carrying *SPAvr1b-pSUC2* and *SPFsRGAE1-pSUC2*, indicating functional secretion of invertase and thereby validating the secretory activity of the FsRGAE1 signal peptide. Further validation was carried out using 2,3,5-triphenyltetrazolium chloride (TTC) to detect extracellular invertase activity. Both the positive control (*SPAvr1b-pSUC2*) and *SPFsRGAE1-pSUC2* induced the reduction of colorless TTC to insoluble, red-colored formazan (TPF), resulting in visible red colony formation, providing direct biochemical evidence of functional invertase secretion and confirming the secretory capability of the FsRGAE1 signal peptide.

### 3.4. FsRGAE1 Protein Localizes to the Cytoplasm and Nucleus

**Figure 4:**
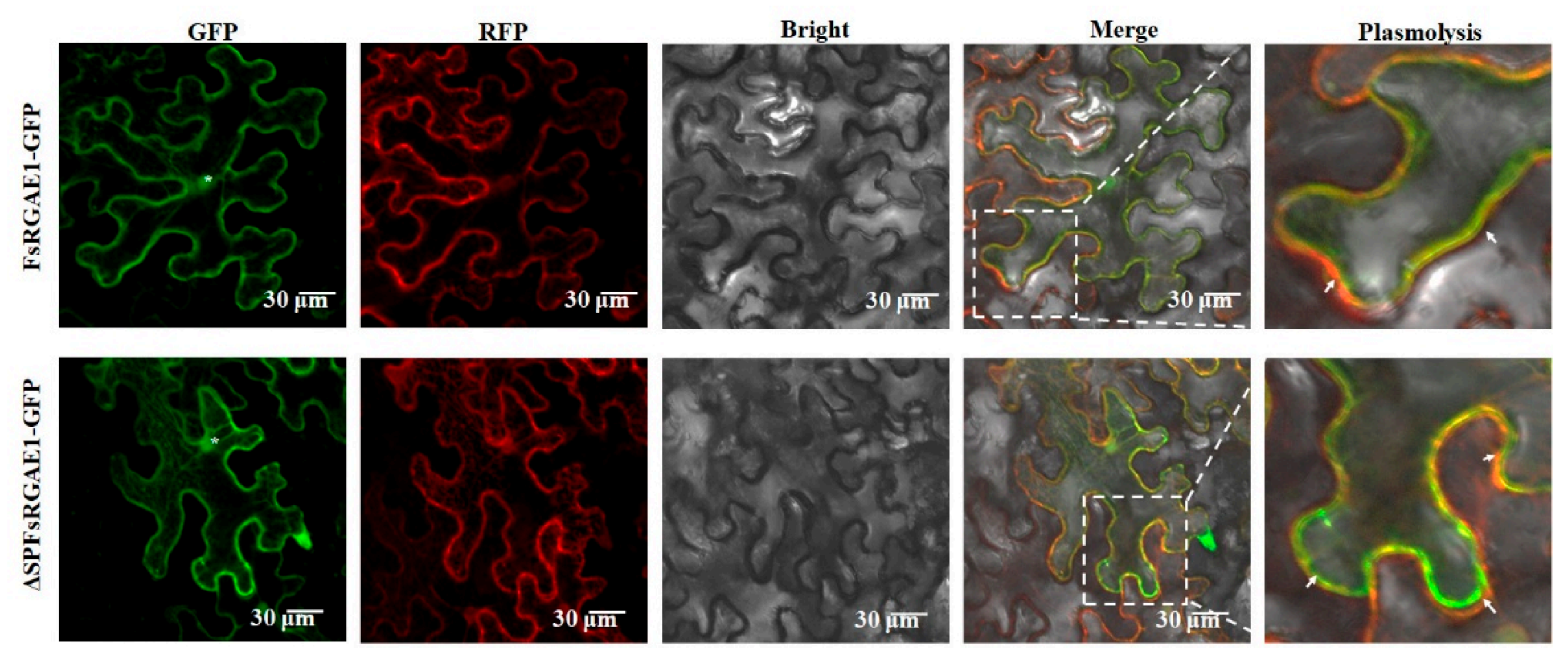
Subcellular localization analysis of FsRGAE in *N. benthamiana* leaves. Representative leaves were taken at 48 h post-inoculation. Representative leaf samples were collected at 48 h post-inoculation. Following plasmolysis induced by treatment with 0.8 M mannitol, GFP or RFP fluorescence signals from FsRGAE1-eGFP, ΔSPFsRGAE1-eGFP, and AtPLC2-RFP (a plasma membrane localization marker) were visualized in epidermal cells using confocal microscopy. The white asterisks denote the nuclei, and the white arrows indicate the retracted plasma membrane. Scale bar = 30 μm.

To further characterize the subcellular localization and biological functions of FsRGAE1, constructs encoding FsRGAE1 with or without its native signal peptide (ΔSPFsRGAE1) were individually fused in-frame to the C-terminus of enhanced green fluorescent protein (eGFP) in the vector pBI121. These constructs were transiently expressed in *N. benthamiana* leaves via *Agrobacterium tumefaciens*-mediated infiltration. GFP fluorescence was monitored 48 h post-infiltration. Subsequent plasmolysis using a 0.8 M mannitol solution, followed by confocal laser scanning microscopy, revealed that FsRGAE1 localizes to both the cytoplasm and nucleus in *N. benthamiana* epidermal cells (Figure 4). In contrast, ΔSPFsRGAE1, lacking the N-terminal signal peptide, exhibited an identical subcellular distribution, indicating that FsRGAE1 constitutively targets the cytoplasm and nucleus in plant cells and that its signal peptide is dispensable for this localization pattern (Figure 4).

### 3.5. FsRGAE1 Contributed to the Pathogenicity of F. sacchari

**Figure 5:**
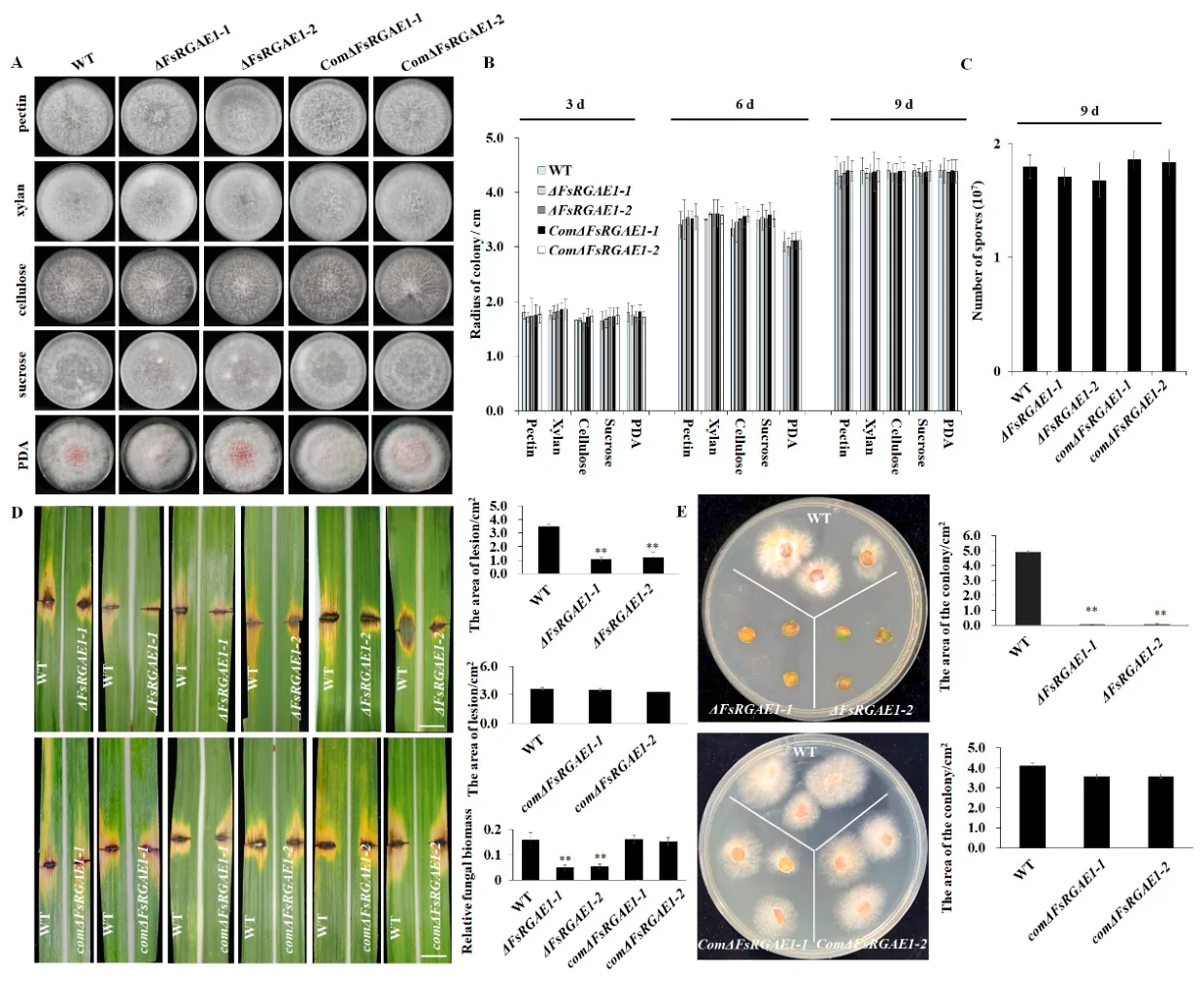
FsRGAE1 contributes to the pathogenicity of *F. sacchari*. (**A**) Vegetative growth and colony morphology of *F. sacchari* wild-type, *ΔFsRGAE1*, and *ComΔFsRGAE1* strains cultured on PDA medium or minimal media supplemented with pectin, xylan, cellulose, or sucrose as the sole carbon source. Photographs were taken after 9 days of incubation. (**B**,**C**) Quantitative analysis of vegetative growth rates (**B**) and conidiation (**C**) for wild-type, *ΔFsRGAE1*, and *ComΔFsRGAE1* strains on PDA or the indicated carbon-source media at 3, 6, and 9 days post-inoculation. Values are the means ± SD; *n* = 3. (**D**) Pathogenicity assays of *ΔFsRGAE1*, and *ComΔFsRGAE1* strains. Disease symptoms on sugarcane leaves inoculated with the wild-type, ∆FsRGAE*1* and *ComΔFsRGAE1* strains on sugarcane leaves were assessed and photographed 7 days post-inoculation. Values are the means ± SD; *n* = 9. The lesion leaves were harvest for relative fungal biomass, and the Values are the means ± SD; *n* = 6. (**E**) Fungal recovery assays from infected leaves. Representative images and quantitative analysis of hyphal recovery from leaves inoculated with wild-type, *∆FsRGAE1* and *ComΔFsRGAE1* strains were performed at 5 days post-inoculation. Values are the means ± SD; *n* = 3. Statistical analysis was performed using Student’s *t*-test. ** *p* < 0.01. Scale bar = 1 cm.

In order to further elucidate the role of FsRGAE1 in regulating virulence of *Fusarium sacchari*, we generated two independent FsRGAE1 knockout mutants (*ΔFsRGAE1-1* and *ΔFsRGAE1-2*) and two complemented strains (*ComΔFsRGAE1-1* and *ComΔFsRGAE1-2*), which were subsequently verified by PCR using both *FsRGAE1*-specific and construct-specific primers, as illustrated in Figure S2. To assess whether FsRGAE1 contributes to fungal growth or carbon source utilization, we cultured the wild-type strain CNO-1, *ΔFsRGAE1*, and the *ComΔFsRGAE1* strains in media containing various carbon sources, and no significant differences were observed among the strains in growth rate and conidiation (Figure 5A–C), indicating that FsRGAE1 is dispensable for vegetative growth and carbon utilization in *F. sacchari*. To investigate the contribution of FsRGAE1 to virulence in the host plant, sugarcane leaves were inoculated with CNO-1, *ΔFsRGAE1*, and *ComΔFsRGAE1* strains. At 7 days post-inoculation, the *ΔFsRGAE1* mutants exhibited significantly attenuated virulence, producing lesions with a markedly smaller area and less fungal biomass than those induced by the wild-type strain (Figure 5D). In contrast, the complemented strain *ComΔFsRGAE1* fully restored pathogenicity, and the lesion area it induced was statistically indistinguishable from that of the wild type (Figure 5E). Concurrently, fungal recovery assays revealed that the ΔFsRGAE1 mutant accumulated significantly less fungal biomass in the host compared with both the wild-type and *ComΔFsRGAE1* strains. These findings collectively demonstrate that FsRGAE1 plays a critical role in the *pathogenicity* of *F. sacchari*.

### 3.6. FsRGAE1 Suppresses the Host’s Defense Response

**Figure 6:**
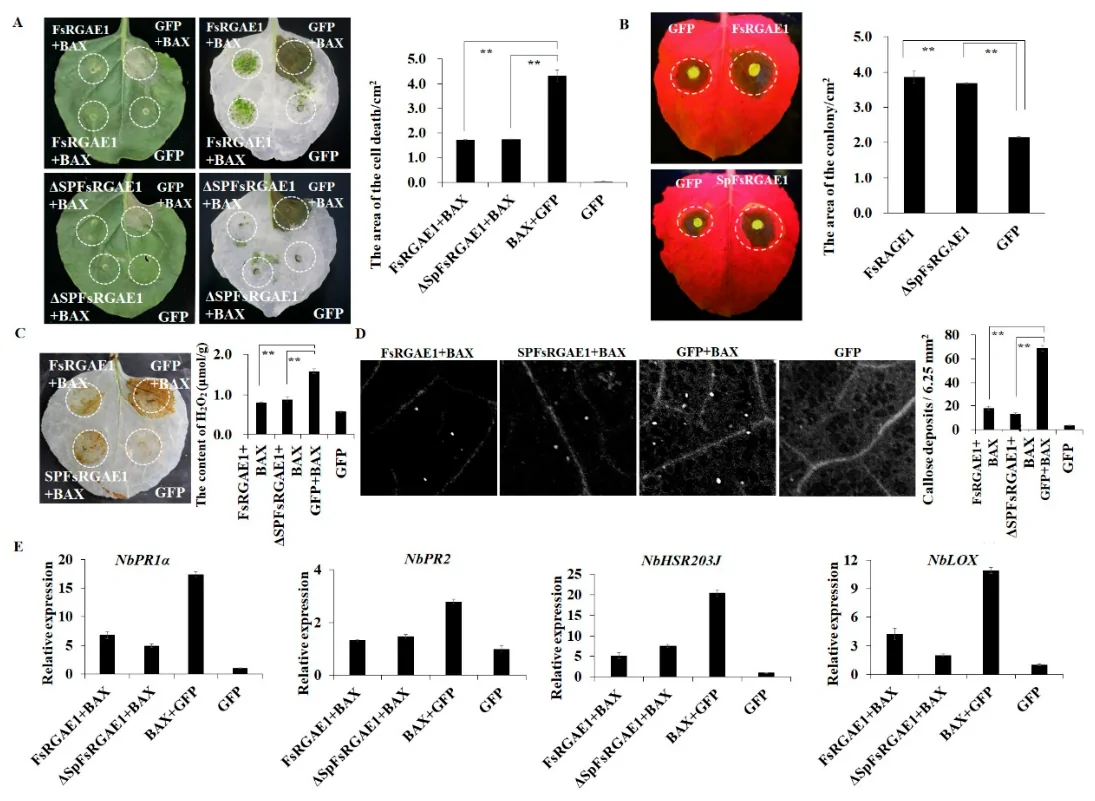
FsRGAE1 suppresses BAX-induced cell death in *N. benthamiana.* (**A**) FsRGAE1 attenuates BAX-triggered programmed cell death in *N. benthamiana*. GFP and BAX served as negative and positive controls, respectively. The cell-death areas of the indicated infiltrated regions were calculated at 5 d post-inoculation. Values are the means ± SD; *n* = 9. (**B**) Transiently expression FsRGAE1or ΔSPFsRGAE1 enhanced *N. benthamiana* susceptibility to *B. cinerea*. Representative photographs were taken at 3 d post *B. cinerea* inoculation. Statistical analysis of the lesion area caused by *B. cinerea* in *N. benthamiana* leaves transiently expressing the indicated constructs were taken at 5 d post *B. cinerea* inoculation. Values are the means ± SD; *n* = 3. (**C**) H_2_O_2_ staining and determination. Values are the means ± SD; *n* = 6. (**D**) Callose staining and determination. Values are the means ± SD; *n* = 3. (**E**) Relative expression levels of defense-related genes in *N. benthamiane* leaves transiently expressing the indicated constructs. Values are the means ± SD; *n* = 3. Statistical analysis was performed using Student’s *t*-test. ** *p* < 0.01.

To better understand the role of FsRGAE1 in mediating multivariate interactions between fungi and their hosts, the full-length coding sequences of *FsRGAE*, *∆SPFsRGAE1*, *GFP*, and *BAX* (Bcl-2-associated X protein) were individually cloned and inserted into the PVX vector pGR107 with a C-terminal 3× HA tag. These constructs were transiently expressed in *N. benthamiana* leaves via *Agrobacterium tumefacien*-mediated infiltration. As shown in Figure 6A, both FsRGAE1 and ∆SPFsRGAE1 effectively suppressed BAX-induced cell death in *N. benthamiana* leaves. Furthermore, leaves expressing FsRGAE1 or ∆SPFsRGAE1 exhibited enhanced susceptibility to *Botrytis cinerea*, developing significantly larger necrotic lesions compared with GFP-expressing control leaves (Figure 6B). To further investigate whether FsRGAE1 modulates additional defense responses beyond its inhibition of BAX-triggered programmed cell death, we assessed the effects of FsRGAE1 and ∆SP-FsRGAE1 expression on canonical immune responses in *N. benthamiana*, including reactive oxygen species (ROS) bursts, callose deposition, and expression of defense-related genes. Consistent with its anti-cell-death activity, both FsRGAE1 and ∆SP-FsRGAE1 significantly suppressed BAX-induced ROS accumulation (Figure 6C) and callose deposition (Figure 6D) in *N. benthamiana* leaves. RT-qPCR analysis further revealed that FsRGAE1 expression markedly downregulated the transcription of multiple defense-related genes in *N. benthamiana* (Figure 6E). Collectively, these findings demonstrate that FsRGAE1 functions as a suppressor of immune responses in *N. benthamiana*.

### 3.7. The Immunosuppressive Function of FsRGAE1 Depends on Its Nuclear Localization

**Figure 7:**
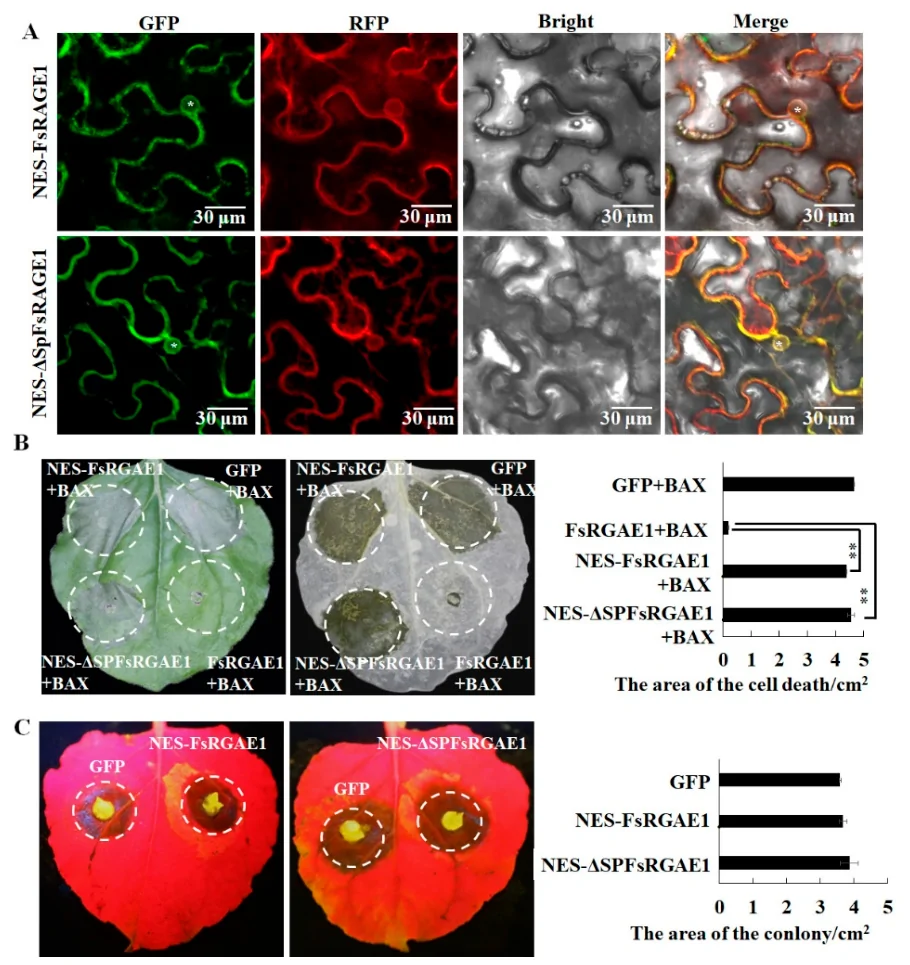
The nuclear localization of FsRGAE1 is essential for its function in suppressing host immunity. (**A**) Subcellular localization of FsRGAE1 and ΔSPFsRGAE1 fused to an N-terminal nuclear export signal (NES) and a C-terminal GFP tag. Representative images were acquired at 60 h post-infiltration. Scale bar = 30 μm. The white asterisks denote the nuclei. (**B**) *N. benthamiana* leaves were separately infiltrated with the indicated *A. tumefaciens* suspensions to detect the function of nucleus-localized FsRGAE1 in manipulating plant immune responses. The cell-death areas of the indicated infiltrated regions were calculated at 5 d post-inoculation. Values are the means ± SD; *n* = 9. (**C**) Lesion diameters on *N. benthamiana* leaves expressing the indicated constructs after *Botrytis cinerea* inoculation, Representative photographs were taken at 3 d post *B. cinerea* inoculation. Values are the means ± SD; *n* = 6. Statistical analysis was performed using Student’s *t*-test. ** *p* < 0.01.

To determine whether FsRGAE1’s suppression of the host immune response is contingent upon its subcellular localization, we fused an N-terminal nuclear export signal (NES) to FsRGAE1 to generate NES-FsRGAE1 and NES-ΔSPFsRGAE1, which were transiently expressed in *N. benthamiana* leaves. As shown in Figure 7A, confocal microscopy revealed that the green fluorescence signal of both NES-FsRGAE1 and NES-ΔSP-FsRGAE1 was nearly absent from the nucleus and instead predominantly accumulated in the cytoplasm. Notably, neither NES-FsRGAE1 nor NES-ΔSPFsRGAE1 suppressed BAX-induced cell death (Figure 7B). Furthermore, *N. benthamiana* leaves expressing NES-FsRGAE1 and NES-ΔSPFsRGAE1 exhibited similar susceptibility to *Botrytis cinerea* as that of GFP, with no significant differences in lesion areas on the leaves compared with that of GFP control (Figure 7C). Collectively, these results demonstrate that FsRGAE1 effectively suppresses immune response in *N. benthamiana* primarily through its nuclear localization within host cells.

## 4. Discussion

Fungal plant pathogens pose substantial economic threats, not only compromising field crop productivity but also inducing postharvest diseases [21]. It is estimated that fungal infections account for approximately 10% of annual global agricultural yield losses [22]. Furthermore, climate change is exacerbating this challenge, with projections indicating that such losses will continue to rise [23]. To mitigate fungal infections, farmers primarily depend on disease-resistant crop varieties or repeated fungicide applications, both of which carry potential environmental risks. Compounding this issue, prevailing agricultural practices emphasize monoculture and the large-scale cultivation of single-genotype crops, which accelerates the selection and proliferation of fungal strains capable of rapidly overcoming host genetic resistance [24]. Consequently, continuous development and deployment of novel resistance traits through breeding programs, as well as advancement of environmentally sustainable and precisely targeted bio-agricultural strategies, are critically needed. However, realizing these objectives hinges on a comprehensive mechanistic understanding of fungal pathogenesis and the intricate molecular dialogue between pathogens and their host plants. Such knowledge underpins the rational design of targeted interventions and the strategic exploitation of plant-intrinsic resistance and susceptibility genes [25].

The capacity to sequence fungal genomes has yielded unprecedented insights into genome composition, architecture, and plasticity, as well as the evolutionary and adaptive mechanisms underpinning fungal biology [26]. Since Dean et al. first reported the genome of the plant pathogen *Magnaporthe oryzae* in 2005 [27], the number of sequenced genomes from plant-pathogenic fungi has expanded dramatically. This surge has catalyzed substantial advances in our understanding of pathogen virulence and has facilitated the systematic identification of virulence-associated genes, particularly effectors. In our previous study, we performed a de novo genome assembly of the *F. sacchari* strain CNO-1 and computationally predicted 316 candidate effector proteins. Many of these candidates are small, secreted proteins lacking identifiable functional domains. Nevertheless, the molecular mechanisms governing the interaction between *F. sacchari* and sugarcane remain poorly understood [9]. In this study, with advancements in information technology and online bioinformatic tools, such as EffectorP v3.0, SignalP v6.0, and TMHMM v2.0, preliminary screening of effectors has become increasingly efficient. FsRGAE1 is one such candidate effector protein, identified as being highly expressed during the early stages of *F. sacchari* infection. Bioinformatic analysis indicates that FsRGAE1 contains a functional signal peptide, lacks a transmembrane domain, shares significant homology with the catalytic domain of known SGNH hydrolases, and is predicted to encode a CE12-family rhamnogalacturonan acetylesterase (RGAE).

RGAE enzymes are broadly classified within the SGHN-hydrolase family as members of the CE12 carbohydrate-active enzyme (CAZyme) family. Enzymes in the CE12 family feature an SGHN hydrolase domain, a conserved GDSL motif that surrounds the nucleophilic serine residue, and a characteristic α/β/α “sandwich” hydrolase fold [28]. Together with their experimentally characterized substrate specificity, these structural and functional features suggest that RGAE may deacetylate the backbone of rhamnogalacturonan I (RGI, a major pectic polysaccharide component of plant cell walls), thereby facilitating subsequent cleavage by RGI-specific lyases and hydrolases [29,30]. Thus, RGAE is likely secreted extracellularly by pathogens to enhance infectivity by promoting the degradation of pectin in the plant cell wall. In our study, deletion of FsRGAE1 had no significant effect on mycelial growth or conidiation, indicating that FsRGAE1 is dispensable for the maturation and developmental processes of *F. sacchari*. However, loss of FsRGAE1 markedly attenuated the virulence of *F. sacchari* and reduced fungal colonization in sugarcane, suggesting that FsRGAE1 plays a critical role in *F. sacchari* pathogenicity (Figure 5). Notably, our study reveals that FsRGAE1 lacks canonical apoplastic targeting signals and instead localizes to both the nucleus and cytoplasm, indicating that its contribution to virulence enhancement and host immune suppression may be independent of its putative SGHN-hydrolase activity (Figure 6 and Figure 7). In this study, we did not assess whether FsRGAE1 possesses enzymatic activity, specifically, whether it exhibits canonical rhamnogalacturonan acetylesterase activity, whether it can catalyze substrates beyond rhamnogalacturonan I (RGI), or whether it modulates immunity through interactions with host proteins; these questions remain open for future investigation.

Transient expression assays in this study revealed that the effector protein FsRGAE1 suppresses BAX-induced cell death and enhances susceptibility to *B. cinerea* in *N. benthamiana*. Moreover, nuclear but not cytoplasm localization of FsRGAE1 is essential for its ability to inhibit the accumulation of key plant defense signals, including callose deposition and reactive oxygen species (ROS), thereby modulating the host immune response. Effectors secreted by pathogens are delivered into distinct subcellular compartments of host cells through diverse secretion systems, thereby suppressing host immune responses and promoting pathogen infection [31]. Based on their site of action, specifically, whether they enter the host cell cytoplasm, effectors are broadly categorized as extracellular or intracellular. These two classes of effector proteins act synergistically to compromise both the physical barriers and chemical defense mechanisms of the host. To date, characterized extracellular effectors are predominantly cell-wall-degrading enzymes (CWDEs), which hydrolyze host cell wall components to facilitate pathogen colonization and systemic spread [32]. In contrast, intracellular effectors target multiple subcellular compartments, including the plasma membrane, nucleus, cytoplasm, chloroplasts, and peroxisomes, to disrupt critical immune signaling components and thereby enhance pathogen virulence [33,34,35]. Consequently, the precise subcellular localization of effector proteins holds a crucial role in plant defense responses; moreover, differences in localization often reflect fundamental distinctions in their molecular mechanisms of action [36]. A similar phenomenon has been observed in *Verticillium dahliae*, where glycoside hydrolase family 7 (GH7) proteins are predominantly characterized by cellobiohydrolase (CBH) and endoglucanase (EG) activities and serve as key enzymatic functions essential for hydrolytic carbohydrate metabolism. Six conserved GH7 proteins were identified in *V. dahliae*; deletion of any individual GH7 gene markedly compromised fungal virulence without affecting in vitro or in planta growth; live-cell imaging demonstrated that all six GH7 proteins are translocated into host plant cells and accumulate in the cytoplasm, where they elicit canonical immune responses, including hypersensitive response (HR)-associated cell death, reactive oxygen species (ROS) burst, and callose deposition, strictly independent of their enzymatic activity [37]. These findings suggest that conventional extracellular virulence factors traditionally thought to act only outside host cells can also enter cells and exert previously unrecognized intracellular functions, though their internalization mechanisms remain unclear. Although FsRGAE1 primarily exerts its immunosuppressive function via nuclear localization, the functional role of its cytoplasmic localization remains unclear. In addition, reports indicate that nuclear-localized, immune-suppressive effectors can suppress host immune responses either by disrupting the normal subcellular localization of their host interaction targets or by directly modulating host defense-related proteins. For instance, FsMEP1, a zinc-dependent metalloprotease effector from *F. sacchari*, suppresses plant immunity and enhances the pathogenicity of *F. sacchari* by re-localizing the chloroplast-localized sugarcane thiamine thiazole synthase ScTHI2 to the nucleus. This mis-localization disrupts the normal subcellular distribution of ScTHI2, thereby impairing thiamine biosynthesis and compromising THI2-mediated defense responses [10]. MoHTR1 and MoHTR2, effector proteins secreted by *Magnaporthe oryzae*, localize to the host nucleus and reprogram the expression of immunity-associated genes by directly binding to the promoters of key immune regulators, including *OsMYB4*, *OsHPL2* and *OsWRKY45* [38]. To fully elucidate the immune-suppressive molecular mechanism of FsRGAE1, comprehensive functional characterization is essential, particularly the identification and experimental validation of its interacting protein partners or DNA targets.

In summary, we identified and functionally characterized a rhamnogalacturonan acetylesterase type effector FsRGAE1 in *F. sacchari*, which exhibits structural features suggestive of a role in plant cell wall degradation. However, contrary to expectations for a secreted cell-wall-modifying enzyme, FsRGAE1 localizes exclusively to the host cytoplasm and nucleus, functioning as an intracellular effector. We demonstrated that FsRGAE1 suppresses host immunity in a nucleus-dependent manner by attenuating ROS accumulation, callose deposition, and the expression of defense related genes in the nucleus-localization manner, thereby enhancing fungal virulence during the initial phase of the *F. sacchari*–sugarcane interaction. Nevertheless, the precise molecular mechanisms underlying FsRGAE1’s function in this pathosystem remain to be fully elucidated and warrant further investigation.

## Data Availability

The original contributions presented in this study are included in this article or in the Supplementary Materials. Further inquiries can be directed to the corresponding author.

## Supplementary Materials

The following supporting information can be downloaded at: https://www.mdpi.com/article/10.3390/jof12090638/s1, Figure S1: Cloning and characterization of *FsRGAE1* from *Fusarium sacchari*; Figure S2: Generation of *FsRGAE1* knockout and complemented mutants in *Fusarium sacchari*; Table S1: Primers used in this study.
