# Supplementary material for: A Rhamnogalacturonan Acetylesterase Effector FsRGAE1 Enhances the Virulence of Fusarium sacchari by Localizing to the Nucleus and Suppressing Plant Immunity
Source: J Fungi (Basel). 2026 Aug 26;12(9):638. doi: 10.3390/jof12090638 (PMC13608016; doi:10.3390/jof12090638)
Supplement: Supplementary file 1 [file jof-12-00638-s001.zip › jof-4462376-supplementary.pdf]

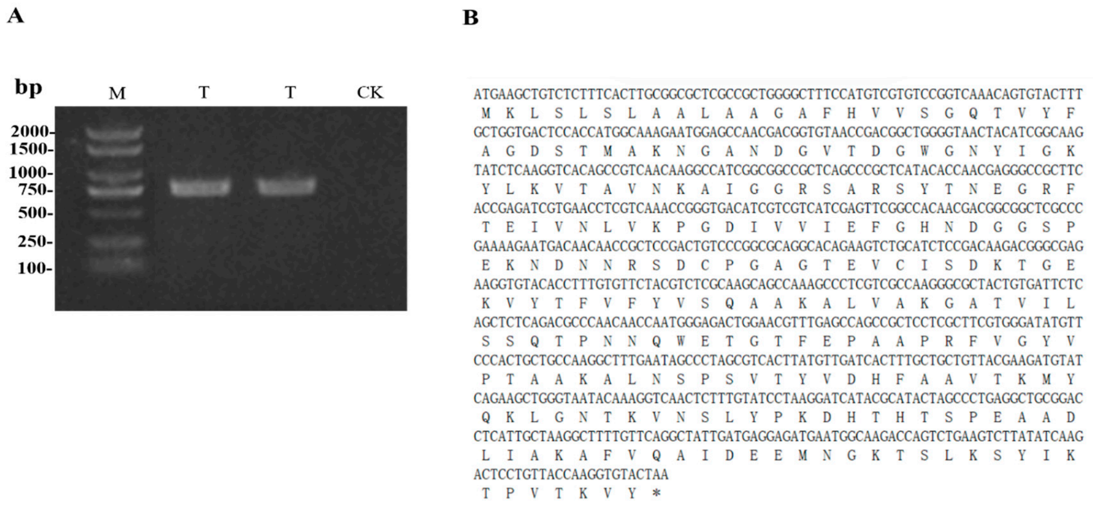

Figure S1. Cloning and characterization of *FsRGAE1* from *Fusarium sacchari.* (A) PCR amplification of *FsRGAE1* coding sequence from DNA of *F. sacchari*. M, DL2000 DNA marker; T, PCR product of Fs06027; CK, ddH2O negative control. (B) A schematic diagram of the protein structure of FsRGAE1. (C) Nucleotide sequence of *FsRGAE1* and its corresponding deduced amino acid sequence.

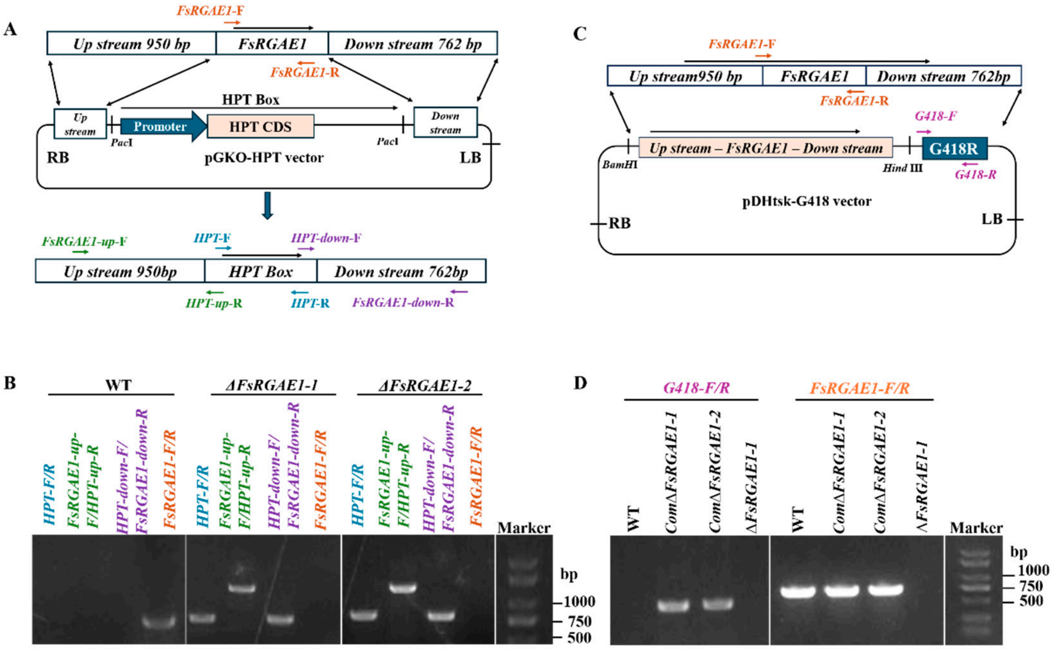

Figure S2. Generation of FsRGAE1 knockout and complemented mutants in *Fusarium sacchari.* (A) Schematic illustration of the strategy employed to generate the *FsRGAE1* knockout mutants (*ΔFsRGAE1*) in *F. sacchari*. (B) PCR-based verification of the *ΔFsRGAE1* strains using the primer pairs indicated in (A). (C) Schematic illustration of the strategy used to construct the *FsRGAE1*-complemented strain (*ComΔFsRGAE1*) in the Δ*FsRGAE1* background. (D) PCR-based verification of the *ComΔFsRGAE1* strains using the primer pairs indicated in (C).

Table S1. Primers used in this study.		
	Primer name	Primer sequence (5' to 3')
RT-qPCR	qFsRGAE1-F	ATGAAGCTGTCTCTTTCACT
	qFsRGAE1-R	TTAGTACACCTTGGTAACAGG
	FvActin-F	GAGAACGAGCGTGTCTTGATTGAGCC
	FvActin-R	TTCCICCGCAGAATGAAGAAGGACTC
	NbEF1α-F	TTGCTTGCTTTCACCCTTGG
	NbEF1α-R	TCAAAACCAGAGATGGGGAC
	NbPR1α-F	CCGCCTTCCCTCAACTCAAC
	NbPR1α-R	GCACAACCAAGACGTACTGAG
	NbPR2-F	CACTGGCACATCATACCTTACC
	NbPR2-R	TCCTAAACGAACCCTGTGATGG
	NbHSR203J-F	GGCAGTGGAGGAGCTTAAAT
	NbHSR203J-R	GCTATGTCCCACTCCATTGTTA
	NbLOX-F	AAAACCTATGCCTCAAGAAC
	NbLOX-R	ACTGCTGCATAGGCTTTGG
	ScGAPDH-F	CACGGCCACTGGAAGCA
	ScGAPDH-R	TCCTCAGGGTTCCTGATGCC
Yeast Signal Sequence Trap System	SPFsRGAE1-pSUC2-F	GGAATTTTAATTAAGAATTCATGAAGCTGTCTCTTTCACT
	SPFsRGAE1-pSUC2-R	CTATAGGGAGAACCTCGAGACCGGACACGACATGGAAAG
Fungal biomass detection	FsITS-F	CGATTACCAGTAACGAGGGTTT
	FsITS-R	ACAACGGATCTCTTGGTTCTG
Subcellular Localization	FsRGAE1-PBI121-F	ACACGGGGGACTCTAGAGGATCCATGAAGCTGTCTCTTTCAC
	ΔSPFsRGAE1-PBI121-F	ACACGGGGGACTCTAGAGGATCCATGCAAACAGTGTACTTTGC
	FsRGAE1-PBI121-R	GCCCTTGCTCACCATGGTACCGTACACCTTGGTAACAGG
Transformation for Gene Deletion and Complementation	FsRGAE1-full-F	AGACAACTCCCGTGCCTCAAC
	FsRGAE1-full-R	TTGGAGTTTGGGAAGGAGACG
	FsRGAE1-pGKO-up-F	AGCTCGCTGAGGGTTTAAATACCAATGCCACATCCTCG
	FsRGAE1-pGKO-up-R	GGCCCGCTGAGGACTTAAGATGACTTGAGTTTCAGATA
	FsRGAE1-pGKO-down-F	ACTAGTGCTGAGGCATTAAGCGTAGTTTGGCCAAGTGTC
	FsRGAE1-pGKO-down-R	AAGCTTGCTGAGGTCTTAACGACATCCGCAGTGCCCTTA
	FsRGAE1-F	ATGAAGCTGTCTCTTTCACT

	FsRGAE1-R	TTAGTACACCTTGGTAACAGG
	HPT-F	ACACTACATGGCGTGATTTCAT
	HPT-R	TCCACTATCGGCGAGTACTTCT
	HPT-up-R	TGAATCTGAAATAAAGGGAGGA
	HPT-down-F	GACTGAGGAATCCGCTCTTGG
	FsRGAE1-up-F	AGACAACTCCCGTGCCTCAAC
	FsRGAE1-down-R	TTGGAGTTTGGGAAGGAGACG
	FsRGAE1-pDHtsk-F	GGTCGACGGTATCGATAAGCTTATACCAATGCCACATCCTCG
	FsRGAE1-pDHtsk-R	GAGGGTTAATTGCGCGGATCCCGACATCCGCAGTGCCCTTA
	G418-F	CTCTGATGCCGCCGTGTTCC
	G418-R	GCGGCGATACCGTAAAGCAC
Agrobacterium-mediated Transient Expression in *N. benthamiana*	NES-FsRGAE1-F	GCTTCGTTTTGAATCGTCGAATGAAGCTGTCTCTTTCAC
	NES-ΔSPFsRGAE1-F	GCTTCGTTTTGAATCGTCGAATGCAAACAGTGTACTTTGC
	NES-FsRGAE1-R	TCGCCCTTGCTCACCATCAGGTACACCTTGGTAACAGG
	FsRGAE1-PVX-F	GCACCAGCTAGCATCGATATGAAGCTGTCTCTTTCAC
	ΔSPFsRGAE1-PVX-F	GCACCAGCTAGCATCGATATGCAAACAGTGTACTTTGC
	FsRGAE1-PVX-R	GTATGGGTACGCGGCCGCGTACACCTTGGTAACAGG
	NES-FsRGAE1-F	GCTTCGTTTTGAATCGTCGAATGAAGCTGTCTCTTTCAC
	NES-ΔSPFsRGAE1-F	GCTTCGTTTTGAATCGTCGAATGCAAACAGTGTACTTTGC
	NES-FsRGAE1/ΔSPFsRGAE1-R	GCTTCGTTTTGAATCGTCGAATGCAAACAGTGTACTTTGC
